# Hybrid *Vibrio vulnificus*

**DOI:** 10.3201/eid1101.040440

**Published:** 2005-01

**Authors:** Naiel Bisharat, Daniel I. Cohen, Rosalind M. Harding, Daniel Falush, Derrick W. Crook, Tim Peto, Martin C. Maiden

**Affiliations:** * University of Oxford, Oxford, United Kingdom; † Tel Aviv University, Ramat Aviv, Israel

**Keywords:** disease outbreaks, emerging, evolution, genetic, hybridization, Israel, marine biology, molecular sequence data, Vibrio vulnificus, research

## Abstract

The recent emergence of the human-pathogenic *Vibrio vulnificus* in Israel was investigated by using multilocus genotype data and modern molecular evolutionary analysis tools. We show that this pathogen is a hybrid organism that evolved by the hybridization of the genomes from 2 distinct and independent populations. These findings provide clear evidence of how hybridization between 2 existing and nonpathogenic forms has apparently led to the emergence of an epidemic infectious disease caused by this pathogenic variant. This novel observation shows yet another way in which epidemic organisms arise.

## Abstract 2

Hybridization between natural populations of *Vibrio vulnificus* results in hyperinvasive clone.

*Vibrio vulnificus*, a ubiquitous inhabitant of marine and estuarine environments, is considered one of the most dangerous waterborne pathogens. The case-fatality rate for *V. vulnificus* septicemia may reach 50% (*1*). Human infection is generally acquired through eating contaminated raw or undercooked seafood or through contamination of wounds by seawater or marine animals (*2*). Infected persons with preexisting liver disease, hemochromatosis, or compromised immune systems are at particularly high risk for fatal septicemia (*3*–*8*).

Human infections are sporadic and almost entirely caused by strains of biotype 1, while biotype 2 strains have been reported to cause disease mainly among eels and rarely infect humans (*9*). During the summer of 1996, a major outbreak of systemic *V. vulnificus* infections started among Israeli fish market workers and fish consumers (*10*,*11*). Molecular studies showed that the disease outbreak was caused by a previously undescribed biotype that exhibited a distinct phenotypic and molecular pattern, designated biotype 3 (*11*).

The origins of this emergent infectious disease have not been fully understood, although it was originally thought to arise mostly from human behavior and work practices (*10*). On the basis of these assumptions, new fish-handling procedures were introduced (*11*,*12*). However, disease continued, although at a lower incidence. Therefore, studies were undertaken to determine whether this novel outbreak of disease was caused by a specific lineage or clone. The emergence of this new biotype could not be resolved by conventional microbiologic and molecular typing approaches. We investigated this outbreak by combining a multilocus sequence typing approach (*13*) with molecular evolutionary analyses.

## Materials and Methods

### Bacterial Isolates

To study the emergence of this new biotype, we examined a collection of 159 *V. vulnificus* isolates that represented all 3 biotypes from human disease and environmental sources that originated in Israel (n = 64), the Untied States (n = 54), Denmark (n = 7), Germany (n = 6), Spain (n = 5), Sweden (n = 5), Japan (n = 8), South Korea (n = 2), Singapore (n = 2), Thailand (n = 1), Indonesia (n = 1), and Taiwan (n = 1). In addition, 3 well-characterized reference strains that represented the 3 biotypes were included, the ATCC 27562 strain (biotype 1, isolated from human blood in the USA), the E-39 strain (biotype 2, isolated from diseased eel in Spain), and ATCC BAA-86 (biotype 3, isolated from human blood in Israel). Biotype 1 strains (n = 82) consisted of 39 isolates from human disease and 43 environmental isolates; biotype 2 strains (n = 15) consisted of 13 isolates from diseased eels, 1 from and infected person, and 1 from diseased shrimp. Biotype 3 strains were isolated from samples from persons with invasive disease in Israel (n = 61) and from fish-pond water (n = 3). Isolates were grown on blood agar plates and incubated overnight at 35°C in aerobic conditions. The lists of the isolates used in this study and their sources can be accessed at http://pubmlst.org/vvulnificus.

### DNA Extraction

The DNeasy kit (QIAGEN GmbH, Hilden, Germany) was used to extract DNA with the gram-negative bacterial protocol as recommended by the manufacturer. Briefly, several colonies from a bacterial culture were picked off into phosphate-buffered saline solution and centrifuged at 7,500 rpm (5,000 x *g*) for 10 min. The cell pellet was resuspended in 180 μL of tissue lysis buffer, then 20 μL of proteinase K (10 mg/mL) was added, and the sample was incubated at 55°C until the tissue was completely lysed. Then 200 μL of lysis buffer was added and incubated at 70°C for 10 min. The DNA in the clear viscous lysates was precipitated with ethanol 95% (vol/vol) and added to DNeasy mini columns. Ethanol 70% (vol/vol)–based buffers (AW1 and AW2) were added sequentially to the columns and centrifuged at 8,000 rpm (6,000 x *g*). The supernatants were discarded, and the DNA was resuspended in AE buffer and used for amplification.

### Multilocus Sequence Typing (MLST)

This bacterium has 2 chromosomes. Fourteen housekeeping genes (7 from each chromosome) that encoded enzymes responsible for intermediary metabolism were identified by searching the genome database (http://www.ncbi.nlm.nih.gov/genomes/MICROBES/Complete.html) of *V. vulnificus* strain CMCP6, with gene sequences from other bacteria. Genetic loci were chosen for further investigation on the basis of the following criteria: chromosomal location, suitability for primer design, and sequence diversity in pilot studies. Ten loci were chosen for the MLST scheme, 5 from each chromosome. The following were chosen from the large chromosome: *glp,* the encoding glucose-6-phosphate isomerase; *gyrB*, the encoding DNA gyrase-subunit B; *mdh*, the encoding malate-lactate dehydrogenase; *metG*, the encoding methionyl-tRNA synthetase; and *purM*, the encoding phosphoribosylaminoimidazole synthetase. The following were chosen from the small chromosome: *dtdS*, the encoding threonine dehydrogenase; *lysA*, the encoding diaminopimelate decarboxylase; *pntA*, the encoding transhydrogenase alpha subunit;; *pyrC,* the encoding dihydroorotase; and *tnaA*, the encoding tryptophanase. Their chromosomal location suggested that it was unlikely for any of the loci to be coinherited in the same recombination event, as the minimum distance between loci was 300 kb (Table).

**Table:** Characteristics of loci included in the *Vibrio vulnificus* MLST scheme*

Locus	Size of sequenced fragment (bp)	No. of alleles identified	No. of polymorphic sites (%)	Position in *V. vulnificus* genome† (bp)
*glp*	480	38	46 (9.6)	Chromsome I (1379280)
*gyrB*	459	31	34 (7.4)	Chromsome I (999145)
*mdh*	489	29	30 (6.1)	Chromsome I (649619)
*metG*	429	31	37 (8.6)	Chromsome I (3091694)
*purM*	444	28	39 (8.8)	Chromsome I (1895474)
*dtdS*	417	46	56 (13.4)	Chromsome II (1621665)
*lysA*	465	41	78 (16.8)	Chromsome II (1110400)
*pntA*	396	32	35 (8.8)	Chromsome II (332656)
*pyrC*	423	35	50 (11.8)	Chromsome II (1752259)
*tnaA*	324	32	42 (12.9)	Chromsome II (926270)

*From the *V. vulnificus* genome (http://www.ncbi.nlm.nih.gov/genomes/MICROBES/Complete.html). †MLST, multilocus sequence typing.

### Amplification and Nucleotide Sequence Determination

Polymerase chain reaction (PCR) products were amplified with oligonucleotide primer pairs designed from the *V. vulnificus* genome sequence. These primers provided reliable amplification from a diverse range of samples (available from http://pubmlst.org/vvulnificus). Each 50-μL amplification reaction mixture was made up of 10 ng of *V. vulnificus* chromosomal DNA, 100 pmol of each PCR primer (MWG Biotech, Ebersberg, Germany), 10 x PCR buffer with 1.5 mM MgCl_2_ (QIAGEN GmbH), 0.5 U of Taq DNA polymerase (QIAGEN GmbH), and 1.6 mM deoxynucleoside triphosphates (ABgene, Epsom, UK). The reaction conditions were denaturation at 94°C for 1 min, primer annealing at 50°C for 45 s and extension at 72°C for 1 min for 30 cycles. The amplification products were purified by precipitation with 20% polyethylene glycol and 2.5 M NaCl (*14*), and their nucleotide sequences were determined at least once on each DNA strand by using internal nested primers (available from http://pubmlst.org/vvulnificus) and ABI PRISM BigDye Terminators v 3.0 Reaction Mix (Applied Biosystems, Foster City, CA) in accordance with the manufacturer’s instructions. Unincorporated dye terminators were removed by precipitation of the termination products with sodium acetate (3 M, pH 5.2) and 95% ethanol, and the reaction products were separated and detected with an ABI PRISM 3730 DNA Analyzer (Applied Biosystems). Sequences were assembled from the resultant chromatograms with the STADEN suite of computer programs and edited to resolve any ambiguities (*15*). For each locus, every different sequence was assigned a distinct allele number in order of identification; these sequences were internal fragments of the gene, which contained an exact number of codons. Each isolate was therefore designated by a 10-integer number (the allelic profile), which corresponds to the allele numbers at the 10 loci in the following order: *glp*, *gyrB*, *mdh*, *metG*, *purM*, *dtdS, lysA*, *pntA*, *pyrC*, and *tnaA*. Isolates with the same allelic profile are assigned to the same sequence type (ST), which were numbered in the order of their identification (ST-1, ST-2, and so on). The data have been deposited (http://pubmlst.org/vvulnificus).

### Inferring the Population Structure and Ancestral Sources

The program STRUCTURE was used to define the population structure and identify the ancestral sources of the 10 gene fragments from all the strains. STRUCTURE is a recently developed program that implements a Bayesian model approach for inferring population structure and ancestral sources from multilocus genotype data (*16*). Of the 4,326 nucleotides sequenced for each isolate from the 10 genes, 447 nt were polymorphic. For the purposes of the analysis, these nucleotides were used by STRUCTURE as individual loci. STRUCTURE can infer the population structure by using a variety of models, including the linkage model (*17*), which incorporates linkage disequilibrium due to correlations in ancestry between loci that reflects admixture between populations. This approach has recently been used to elucidate the structure and evolution of populations of the human pathogen *Helicobacter pylori* (*18*), and we have used the same method here. For the purposes of the analysis, the nucleotide sequence of the 10 housekeeping gene fragments of 2 clinical strains of *V. vulnificus*, CMCP6 and YJ016*,* whose complete genome sequence has been recently completed (http://www.ncbi.nlm.nih.gov/genomes/MICROBES/Complete.html), were added to all the data sets.

## Results

### Genotypes Identified

The 159 isolates were resolved into 70 STs, 56 of which were present only once in the entire collection. Eighty-two isolates (51.6%) were represented by 1 of 4 STs: ST-8 was the most common and occurred 62 times (39%); ST-6 occurred in 11 isolates (6.9%); ST-32 occurred in 5 isolates (3.1%); ST-16 occurred in 4 isolates (2.5%). The remaining 21 isolates resolved into 10 sequence types. Strains of biotype 1 (n = 82) resolved into 66 STs. Biotype 2 (15 isolates) resolved into 4 STs; ST-6, ST-9, ST-10, and ST-48. ST-6 was the most common, occurring 11 times and consisting of all the indole-negative isolates. All biotype 3 strains (n = 62) were genetically identical and belonged to ST-8.

### Population Structure and Ancestral Sources

**Figure 1:**
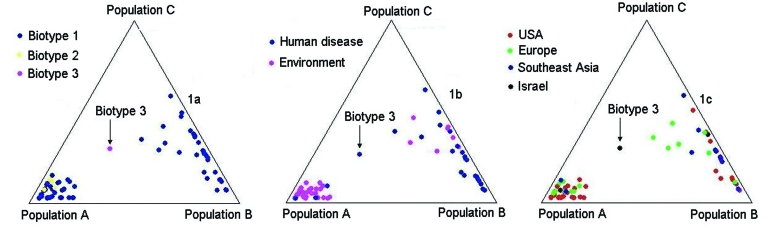
Triangle plots of STRUCTURE results. Only 2 populations were identified (A and B). Figure 1A shows the distribution of the biotypes within the 2 populations. B shows the distribution of the strains according to their source (human or environmental). C shows the strains distribution according to their geographic origin. These results were produced by the linkage model of STRUCTURE with K = 3.

The observed sequence variation between the 2 chromosomes was comparable. Initial analysis of sequence data from the 10 gene fragments showed that extensive recombination had occurred within all the genetic loci under study (data not shown). Constructing phylogenetic trees in the presence of recombination is problematic because different parts of the sequence may have different phylogenetic histories. Therefore, we analyzed the data with the program STRUCTURE. First, we tested the assumption that the 3 *V. vulnificus* biotypes represent 3 distinct predetermined populations of this pathogen (K = 3). The results of multiple analyses with STRUCTURE were incompatible with this assumption; in all cases, only 2 populations were identified, populations A and B (Figure 1 and data not shown). Further, while biotype 1 was present in both populations, biotype 2 was present only in population A (Figure 1A). Biotype 3 occupied an intermediate position between the 2 populations (Figure 1A). Figure 1B shows that an overrepresentation of human disease isolates occurred in population B and an overrepresentation of environmental isolates occurred in population A. And Figure 1C shows that both populations were globally distributed.

**Figure 2:**
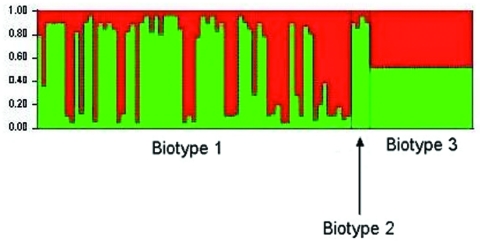
Results of a Bayesian cluster analysis by STRUCTURE. Each of the strains included in the analysis is represented by a thin vertical line, partitioned into 2 colored segments that represent the proportion of polymorphic sites inherited from each of the 2 genetic ancestries. For the representation of results, strains were grouped according to biotype. The analysis was carried out by using the linkage model with K = 2.

**Figure 3:**
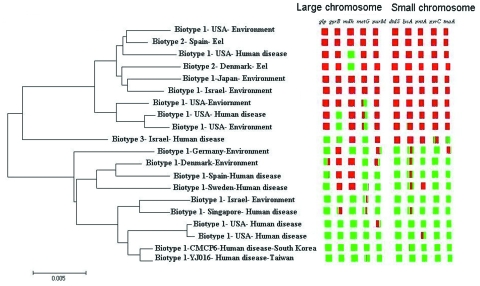
A neighbor-joining tree of representative isolates from the 2 populations is plotted with the inferred ancestral sources of individual polymorphic sites in each of the 10 genes. The names of the genes and to which chromosome they belong are indicated. These results are based on using the linkage model of STRUCTURE with K = 2.

To identify the evolutionary processes underlying the emergence of the genotype responsible for the Israeli outbreak, we repeated the STRUCTURE analysis assuming only 2 populations (K = 2) (based on the findings from the first STRUCTURE analysis). This analysis identified the ancestral sources of the individual strains (Figure 2). Each strain is represented by a thin vertical line partitioned into 2 (K = 2) most likely predetermined populations or genetic ancestries. Each line shows the proportion of polymorphic sites inherited from each of the 2 populations (shown in green and red). It shows that most biotype 1 and 2 strains have predominant contribution from 1 of the 2 genetic ancestries. However, strains of biotype 3 have almost equal contributions from both genetic ancestries. This analysis was further detailed to identify the ancestral sources of each of the polymorphic sites in each of the 10 gene fragments (Figure 3).These analyses confirmed that, notwithstanding the subdivision of *V. vulnificus* populations into 2 populations, recombination had occurred between these populations and that the Israeli outbreak genotype is a hybrid, with some genes originating from 1 population and some from another, while some genes have representation from both.

## Discussion

We have shown that a hybrid virulent organism that acquired genes from 2 distinct and independent populations has caused the disease outbreak in Israel. To achieve this analysis, we studied large, carefully assembled, collections of *V. vulnificus* isolates. The human strains were collected from infected patients in Israel, the United States, Europe, and Southeast Asia. The environmental strains were collected from environmental sources in the United States, the Pacific Ocean, the Baltic Sea, inland fish farms in Israel, and eel farms in Europe.

The division of *V. vulnificus* populations into 2 major groups is consistent with results of multilocus enzyme electrophoresis studies (*19*). However, those studies placed the Israeli electrophoretic type within 1 of the 2 groups, in contrast to our findings, which placed the Israeli genotype in an intermediate position between the 2 populations (Figure 1A and Figure 3).

Hybridization within bacterial populations, i.e., the process whereby a hybrid results from the hybridization of the genomes of 2 or more populations of a species or an organism, has been the focus of much attention by scientists in the last decade (*20*–*25*); these events, which may be intra- or interspecies, could alter the genetic distances and the phylogenetic relationships within bacterial populations. The magnitude by which these events occur is crucially dependent on ecologic factors; different populations of a species must be present within the same niche for genetic exchange to have an impact on genetic variation (*26*). Multiple sampling of fish-farm water and fish documented the abundance of biotype 1 strains (11 and data not shown). These biotype 1 strains, representing both populations of *V. vulnificus*, were never implicated in disease among fish-farm fish, according to the Central Fish Health Laboratory, Kibbutz Nir David, Israel (www.moag.gov.il/english), or among humans in Israel (*11*). These observations are consistent with finding that these populations are not pathogenic to either humans or fish. The finding that this hybrid variant (biotype 3) was the only implicated organism in all disease cases from 1995 to 2003 is indicative of its pathogenicity. Furthermore, the finding that all 62 biotype 3 strains were genetically identical could suggest that this hybrid clone may have evolved by a relatively recent genome hybridization event.

Hybrid variants have been recently described among populations of *Staphylococcus aureus* (*27*) and *Chlamydia trachomatis* (*28*). However, our findings show the first bacterial variant that is clearly more pathogenic than the existing forms of the organism, i.e., the Israeli hybrid clone, is more pathogenic than the existing biotype 1 strains within the Israeli aquaculture system. These findings are consistent with observations among influenza viruses (*29*). This phenomenon has also recently been described also among populations of mosquitoes (*30*), in which hybridization between existing forms of a relatively nonpathogenic organism has apparently led to the emergence of a novel pathogenic variant that poses a particular threat to human health.

The Israeli genotype spread extensively after its emergence in 1995, and by 2003, most of the fish farms in Israel were the sources of *V. vulnificus* cases. This finding is consistent with the idea that this pathogen is circulating freely within the underground brackish water reservoirs that supply these fish farms. Despite the widespread use of inland fish farming around the world, no similar outbreaks have been reported. During the 1970s and 1980s, the introduction into Israel of stocks of *Tilapia* spp. from Africa, the Far East, and South America (*31*–*33*) (for experimental and commercial purposes) may have contributed to the evolution of this hybrid clone. In view of the widespread fish-trading industry, this hybrid clone may eventually emerge through exports of Israeli tilapia stocks, in remote geographic locations. In conclusion, these observations demonstrate the power of molecular and population genetic approaches in investigating the emergence of a novel pathogen and defining its nature. Our results show another way by which epidemic infectious diseases arise.
